# Subcellular localization of Arabidopsis arogenate dehydratases suggests novel and non-enzymatic roles

**DOI:** 10.1093/jxb/erx024

**Published:** 2017-03-10

**Authors:** Crystal D. Bross, Travis R. Howes, Sara Abolhassani Rad, Ornela Kljakic, Susanne E. Kohalmi

**Affiliations:** 1 Department of Biology, Western University, 1151 Richmond Street North, London Ontario, N6A 5B7, Canada

**Keywords:** Arogenate dehydratase, chloroplast division, moonlighting proteins, nuclear localization, phenylalanine biosynthesis, stromules.

## Abstract

Arogenate dehydratases (ADTs) catalyze the final step in phenylalanine biosynthesis in plants. The *Arabidopsis thaliana* genome encodes a family of six ADTs capable of decarboxylating/dehydrating arogenate into phenylalanine. Using cyan fluorescent protein (CFP)-tagged proteins, the subcellular localization patterns of all six *A. thaliana* ADTs were investigated in intact *Nicotiana benthamiana* and *A. thaliana* leaf cells. We show that *A. thaliana* ADTs localize to stroma and stromules (stroma-filled tubules) of chloroplasts. This localization pattern is consistent with the enzymatic function of ADTs as many enzymes required for amino acid biosynthesis are primarily localized to chloroplasts, and stromules are thought to increase metabolite transport from chloroplasts to other cellular compartments. Furthermore, we provide evidence that ADTs have additional, non-enzymatic roles. ADT2 localizes in a ring around the equatorial plane of chloroplasts or to a chloroplast pole, which suggests that ADT2 is a component of the chloroplast division machinery. In addition to chloroplasts, ADT5 was also found in nuclei, again suggesting a non-enzymatic role for ADT5. We also show evidence that ADT5 is transported to the nucleus via stromules. We propose that ADT2 and ADT5 are moonlighting proteins that play an enzymatic role in phenylalanine biosynthesis and a second role in chloroplast division or transcriptional regulation, respectively.

## Highlight

We show subcellular localization of all six *Arabidopsis thaliana* arogenate dehydratases to chloroplasts and stromules. Furthermore, ADT2 and ADT5 have additional localization patterns suggesting non-enzymatic functions and roles as moonlighting proteins.

## Introduction

**Fig. 1.**
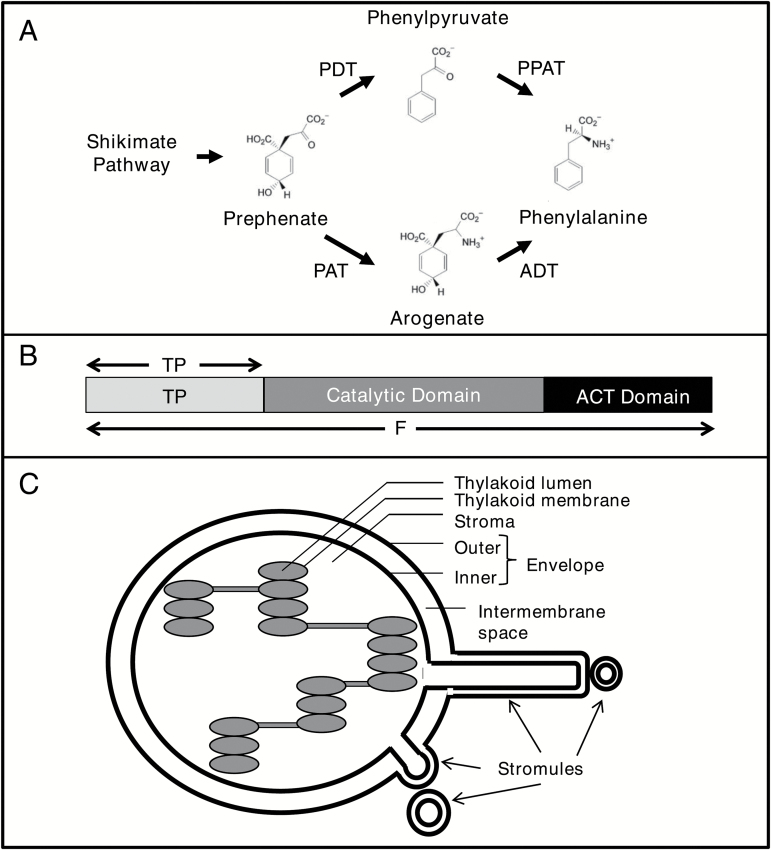
Phenylalanine synthesis, arogenate dehydratases, and stromules. (A) Phenylalanine can be synthesized in plants using either the prephenate (top) or the arogenate (bottom) pathway (Cho *et al.*, 2007; Maeda and Dudareva, 2012). Prephenate is either decarboxylated/dehydrated to phenylpyruvate (PP) by a prephenate dehydratase (PDT) and PP is then transaminated by a phenylpyruvate aminotransferase (PPAT) to phenylalanine. Alternatively the two enzymatic steps are reversed, whereby prephenate is transaminated to arogenate by a prephenate aminotransferase (PAT) and arogenate is then decarboxylated/dehydrated to phenylalanine by an arogenate dehydratase (ADT). (B) *A. thaliana ADT* constructs were cloned in different lengths. The full-length (F) sequence represents the entire *ADT* ORF while an N-terminal construct only includes the transit peptide (TP). (C) Schematic diagram of a chloroplast showing the formation of stromules. Stromules are stroma-filled protrusions of the outer and inner membrane from chloroplasts. They can differ in length, forming long thread-like extensions or globular structures.

Arogenate dehydratases (ADTs; EC 4.2.1.911) catalyze the final step of phenylalanine biosynthesis through decarboxylation/dehydration of arogenate to form the aromatic amino acid phenylalanine (Fig. 1A; Laskar *et al.*, 2010; Tzin and Galili, 2010). In plants phenylalanine serves as a precursor not only of proteins but also of many secondary metabolites, including phenylpropanoids (Herrmann and Weaver, 1999; Knaggs, 2003; Vogt, 2010). Phenylpropanoids have diverse functions, including structural support, pigmentation, and scent formation (Vogt, 2010), indicating the great importance of phenylalanine biosynthesis in plants.

The *Arabidopsis thaliana* genome encodes a small gene family of six *ADT* genes, all sharing similar sequences and domain structures (Ehlting *et al.*, 2005; Cho *et al.*, 2007). Families of ADTs are common in plant genomes and have been identified in both monocot and dicot species (Tuskan *et al.*, 2006; Yamada *et al.*, 2008; Maeda *et al.*, 2010, 2011). The presence of multiple isoforms suggests that ADTs might have evolved different properties, and/or that each ADT is either transcriptionally or post-translationally regulated to allow for distinct functional roles. For example, all six *A. thaliana* ADTs accept arogenate as a substrate, as their name suggests, but two of the six ADTs also accept prephenate [meaning they can act as ADTs and prephenate dehydratases (PDTs; Fig. 1A; Cho *et al.*, 2007; Bross *et al.*, 2011)]. Furthermore, ADTs differentially contribute to lignin content and bolting/flowering transition. Analysis of different *A. thaliana* ADT knockout mutants has indicated that specific ADTs preferentially contribute to the synthesis of different downstream products of the phenylpropanoid pathway (Corea *et al.*, 2012*b*). In lines harboring an adt5 knockout, the amount of phenylalanine, and its proportions relative to tyrosine and tryptophan, were lower in stem tissues compared with the wild-type control (Corea *et al.*, 2012*a*). Together, these data suggest that ADT5 plays a predominant role in phenylalanine biosynthesis for lignin deposition in stems. Also, the *adt1*, but not the *adt4*, knockout line exhibited a resistant late bolting/flowering phenotype compared with wild-type *A. thaliana* under different environmental conditions, which is consistent with a decreased level of *ADT* expression after cold treatment in resistant late bolting/flowering *Beta vulgaris altissima* (Hébrard *et al.*, 2013). This suggests that ADTs may be functional targets of DNA methylation in the shoot apical meristem during vernalization, and that the accumulation of phenolic compounds may play a role in floral transition.

Plant ADTs, including all six *A. thaliana* isoforms, have three domains (Fig. 1B), a putative N-terminal transit peptide (TP), an internal catalytic domain, and a C-terminal ACT (aspartokinase–chorismate mutase–TyrA) domain (Cho *et al.*, 2007). Both the catalytic and ACT domains are conserved across plant, bacterial, and fungal ADTs and PDTs, with the catalytic domain decarboxylating/dehydrating prephenate and/or arogenate (Cho *et al.*, 2007; Bross *et al.*, 2011) while the ACT domain is involved in allosteric regulation induced by ligand binding (Tan *et al.*, 2008; Vivan *et al.*, 2008). The N-terminal domain is unique to plant ADTs and is not found in the bacterial or fungal proteins. In *A. thaliana* ADTs, this domain is ~100–130 amino acids in length, and are likely to be chloroplast TPs according to sequence prediction programs, which is consistent with phenylalanine biosynthesis occurring in chloroplasts (Jung *et al.*, 1986; Cho *et al.*, 2007; Li and Chiu, 2010) and a chloroplastic localization identified for ADTs in protoplasts (Rippert *et al.*, 2009).

Identifying the subcellular localization of proteins can help to define their functional role and has subsequently led to the identification of new unexpected roles (Sparkes and Brandizzi, 2012). This approach can be particularly helpful when dissecting and differentiating the biological roles of members within protein families (Karve *et al.*, 2008). In this study, we provide evidence that most *A. thaliana* ADTs are targeted to the stroma and stromules (stroma-filled tubules; Köhler and Hanson, 2000) of chloroplasts and we show that this targeting is dependent on the presence of the TP. This subcellular localization is consistent with the enzymatic role of ADTs in phenylalanine biosynthesis and the proposed role of stromules in increasing metabolite transport (Natesan *et al.*, 2005). In addition, we demonstrate that two of the ADTs, ADT2 and ADT5, have additional subcellular localization patterns that suggest novel, non-enzymatic functions.

## Materials and methods

### Growth conditions for bacteria and plants

*Escherichia coli* DH5α and DH10β strains (Invitrogen catalog nos 11319019 and 18290015, respectively) were used for the maintenance and amplification of plasmid DNA. *Agrobacterium tumefaciens* strain LBA4404 containing Ti helper plasmid pAL4404 (NCCB accession no. PC2760; Hoekema *et al.*, 1983; Hellens *et al.*, 2000) was used for transformation of *Nicotiana benthamiana* and *A. thaliana*. *Agrobacterium tumefaciens* strain GV3101 (Koncz and Schell, 1986; Hellens *et al.*, 2000) was used for the transformation of the *dominant negative myosin XI-2* (*dnMyoXI-2*) and *dnMyoXI-K/GTD* constructs (Avisar *et al.*, 2008). *Escherichia coli* and *A. tumefaciens* were grown at 37°C in LB medium, and at 28°C in YEB medium, respectively (Vervliet *et al.*, 1975; Bertani, 2004), with media supplemented with appropriate antibiotics.

Three- to five-week-old *N. benthamiana* were used for localization studies of *A. thaliana* ADTs and grown in incubators (Conviron) under 16 h light (80–100 µmol m^−2^ s^−1^) and 8 h dark, with light and dark temperatures set to 24°C and 22°C, respectively. *Arabidopsis thaliana* accession Columbia-0 (Col-0) was grown for 3–4 weeks with the same photoperiod and a light intensity of 150 µmol m^−2^ s^−1^. *Arabidopsis thaliana* plants grown for transient transformations were watered with a 20 mM l-ascorbic acid solution.

The *adt2-1D* mutation is an ethyl methanesulfonate-induced point mutation that causes a serine to be replaced by an alanine in the ACT regulatory domain, leading to an enzyme that is unable to respond to phenylalanine-mediated allosteric inhibition (Huang *et al.*, 2010).

### Cloning of ADT–CFP fusion constructs

Primers were designed (Table 1) to amplify full-length *A. thaliana ADT* genes (*ADT1*, *At1g11790*; *ADT2*, *At3g07630*; *ADT3*, *At2g27820*; *ADT4*, *At3g44720*; *ADT5*, *At5g22630*; and *ADT6*, *At1g08250*) (Ehlting *et al.*, 2005; Cho *et al.*, 2007) and tested (Lynnon BioSoft, Version 6). Amplified full-length *ADT* sequences contain the entire ORF, including the coding sequence for TPs, ADT/PDT catalytic and ACT regulatory domains. In addition, ADT2 was cloned as the TP sequence only (Fig. 1B). Primers were designed to include restriction enzyme cleavage and docking sites, to allow for directional integration of PCR fragments into the target vector (Table 1). *ADT* sequences were amplified with Platinum *Taq* Polymerase High Fidelity (Invitrogen catalog no. 11304011) with previously cloned *ADT* sequences as templates (Cho *et al.*, 2007).

**Table 1.** List of primer sequences

**Name** ^***a***^	**Sequence (5'–3'**)^***b***^	**Restriction enzyme recognition sequence**
CFP-For	*AT* **CGGACCG**GTCGCCACC ATGGTGAGCAAGG	*Cpo*I
CFP-Rev	*TCA* **TCTAGA**TTACTTGTA CAGCTCGTCC	*Xba*I
CFP-Seq	GATCTGAGCTACACATGC	N/A
ADT1-F	**AAGCTT**ATGGCTCTGAGGTGTTTTC	*Hin*dIII
ADT1-R	**GGATCC**TGTCTGACTAGATCCATTGG	*Bam*HI
ADT2-F	**AAGCTT**ATGGCAATGCACACTGTTCG	*Hin*dIII
ADT2-S	**AAGCTT**ATGCGTGTTGCGT ATCAGGGAGTACG	*Hin*dIII
ADT2-R	**GGATCC**AAGAGCATTGTA GTGTCCACTGG	*Bam*HI
ADT2-RTP	**GGATCC**TTAACGCGGGAGCCATTAG	*Bam*HI
ADT3-F	**GAATTC**ATGAGAACTCTCTTACCTTC	*Eco*RI
ADT3-R	**GGATCC**ATCAATGAAAATGTTGATGACG	*Bam*HI
ADT4-F	**CTCGAG**ATGCAAGCCGCAACGTCG	*Xho*I
ADT4-R	**GGATCC**AATGCTTCTTCT GTGGATGTCATGG	*Bam*HI
ADT5-F	**CTCGAG**ATGCAAACCATTTCGCC	*Xho*I
ADT5-R	**CCCGGG**TTACGTCTTCGCTAG	*Sma*I
ADT6-F	**GAATTC**ATGAAAGCTCTATCATC	*Eco*RI
ADT6-R	**GGATCC**ATCGATGAAGTTGATG	*Bam*HI

^*a*^ CFP-For/Rev, amplify cerulean cyan fluorescent protein sequence; CFP-Seq, pCB sequencing primer; F, complementary to the 5' end of the full-length *ADT* coding sequence; S, complementary to the 5' end of the catalytic domain; R, reverse primer complementary to the 3' end of the *ADT* coding sequence; RTP, reverse primer complementary to the 3' end of the transit peptide.

^*b*^ Italics, restriction enzyme docking sites; bold, restriction enzyme recognition sequence, underline, nucleotides to maintain frame; dotted underline, pEZT-NL vector sequence; double underline, introduced start or stop codon; unformatted, original, unmodified template sequence.

The T-DNA binary vector pEZT-NL (Carnegie Cell Imaging Project, http://deepgreen.stanford.edu, last accessed 13 February 2017) was used in conjunction with the pAL4404 Ti-helper plasmid for expression *in planta*. *ADT* genes expressed from pEZT-NL are under the control of the *Cauliflower mosaic virus* (CaMV) *35S* promoter and are translated as C-terminally tagged enhanced green fluorescent protein (EGFP) fusions. For co-localization studies, pEZT-NL was modified by replacing *EGFP*, flanked by *Cpo*I and *Xba*I sites, with the cerulean cyan fluorescent protein (CFP)-coding sequence (Conley *et al.*, 2009*b*). Both PCR-amplified *CFP* sequences and the empty pEZT-NL were double-digested with *Cpo*I and *Xba*I; the resulting fragments were ligated and then transformed into *E. coli*. Positive transformants were selected on LB medium containing gentamicin. The resulting vector was renamed pCB. Using the appropriate restriction enzymes, *ADT* genes were cloned into pCB, and all resulting pCB-ADT vectors were sequenced to ensure proper fusion and sequence integrity of the *ADT–CFP* sequences.

To clone the native *ADT5* promoter (proNat5), 1 kb upstream of the *ADT5* start codon was PCR amplified with primers that added a 5' *Mau*BI and a 3' *Xho*I restriction site. The amplified *Mau*BI–*Xho*I fragment was used to replace the CaMV *35S* promoter in pCB-ADT5, generating the vector proNat5::ADT5:CFP.

### Cloning filamentous temperature sensitive Z2 (FtsZ2)–yellow fluorescent protein (YFP)

The Gateway-compatible vector pLIC6 encoding *FtsZ2-1* cDNA was obtained from the ABRC (stock number DKLAT2G36250; Popescu *et al.*, 2007). Restriction digest of pLIC6 with *Hin*dIII yielded a 2329 bp restriction fragment containing *FtsZ2-1* flanked by *attachment*B (*att*B) sites. This gel-purified fragment was then recombined into pDONR221 (Hartley *et al.*, 2000). The entry clone created was digested with *Ase*I and the expected 2608 bp fragment encoding *FtsZ2-1* flanked by *att*L sites was isolated and recombined into the destination vector pEarleygate101 (Earley *et al.*, 2006), generating an *FtsZ2–YFP* fusion construct with expression regulated by the CaMV *35S* promoter.

### Bacterial transformations

Plasmid DNA was isolated from overnight *E. coli* cultures using an alkaline lysis method (modified from Ish-Horowicz and Burke, 1981; Sambrook and Russell, 2001) and transformed into electrocompetent *E. coli* (ElectroMax DH5α, Invitrogen), or electrocompetent *A. tumefaciens* (Wise *et al.*, 2006), using the Gene Pulser II System (Bio-Rad) set to 2.0 kV, 25 µF capacitance, and 200 Ω or 400 Ω resistance, respectively. Immediately following electroporation, *E. coli* and *A. tumefaciens* cells were incubated for 1 h in non-selective LB medium, before plating on selective medium. Correct insertion of amplicons into plasmid DNA of positive *E. coli* transformants was confirmed by restriction enzyme digestion and sequencing of isolated plasmid DNA. pCB-ADTs were transformed into *A. tumefaciens* LBA4404 containing Ti-helper plasmid pAL4404 (Hoekema *et al.*, 1983; Hellens *et al.*, 2000).

### Organelle markers

To identify stromules, the TP of the small subunit of tobacco RuBisCO fused to YFP (TP-ssRuBisCO–YFP; Nelson *et al.*, 2007) was used in co-localization experiments. This fusion construct is under the control of a CaMV *35S* promoter and, after translation, the TP guides the fusion protein to the chloroplast stroma where it can be used to identify stromules (Nelson *et al.*, 2007).

The T-DNA-containing binary vector pEarleygate301-YFP, encoding *A. thaliana NUCLEOPORIN1* fused to *YFP* (*NUP1–YFP*) is under the expression of its native promoter (Lu *et al.*, 2010) and was used as a nuclear marker.

*Agrobacterium tumefaciens* GV3101 containing pCB302 encoding the dominant negative form of either *N. benthamiana* myosin XI-2 or myosin XI-K/GTD was used to inhibit stromule formation (Avisar *et al.*, 2008; Natesan *et al.*, 2009). Each construct encodes the globular tail domain of the corresponding myosin XI. Expression of the dominant negative constructs in pCB302 is regulated *in planta* by the nopaline synthase promoter (Xiang *et al.*, 1999; Avisar *et al.*, 2008).

### Agroinfiltration of tobacco leaves

*Nicotiana benthamiana* and *A. thaliana* were transiently transformed by pressure-infiltrating cultures of *A. tumefaciens* (Wroblewski *et al.*, 2005; Wydro *et al.*, 2006; Conley *et al.*, 2009*b*). *Agrobacterium tumefaciens* were grown overnight in 3 ml of YEB medium with the appropriate antibiotics. Then 50 μl of the overnight culture was transferred to 50 ml of YEB containing 25 μl of 200 mM acetosyringone and 500 μl of 1 M MES, and grown until cell density reached an OD_600_ of 0.5–0.8. Cells were collected by centrifugation, resuspended in Gamborg’s solution to a final OD_600_ of 1.0, and incubated for an additional hour prior to infiltration. For co-infiltration, equal volumes of *A. tumefaciens* cultures containing different vectors were combined to maintain a final OD_600_ of 1.0. The p19 vector encodes a 19 kDa protein from *Tomato bushy stunt virus*, which has been shown to enhance transgene expression through suppression of post-transcriptional gene silencing (PTGS; Silhavy *et al.*, 2002; Voinnet *et al.*, 2003) and was added to all transient transformations.

A minor variation of this protocol was used for co-expression with *TP-ssRuBisCO–YFP* as this construct produced a very strong fluorescence signal compared with that of ADT–CFP. Therefore, *A. tumefaciens* strains containing *ADT–CFP* and p19 constructs (1:1) were infiltrated 1 d before the infiltration of *TP-ssRuBisCO–YFP* and p19 (1:1) constructs. In addition, *A. tumefaciens* containing the *TP-ssRuBisCO–YFP* construct were infiltrated at a lower OD_600_ of ~0.5. Hence visualization of subcellular localization was performed 4 days post-infiltration (dpi) with *TP-ssRuBisCO–YFP* (which equals 5 dpi, with an *ADT–CFP*).

Transient transformants were assayed 5 dpi using a Leica SP2 confocal laser scanning microscope equipped with a ×63 water immersion objective. The abaxial surface of leaf tissue was viewed to observe the localization pattern of fluorescent proteins in the lower epidermis and in mesophyll cells. CFP and chlorophyll were excited with a blue diode laser (405 nm), and emission was collected from 440 nm to 485 nm and from 630 nm to 690 nm, respectively. YFP was excited with a 514 nm argon laser and its emission was collected from 540 nm to 550 nm. For co-localization experiments, CFP and YFP emissions were collected sequentially to avoid emission crosstalk of the fluorophore pair (Shaner *et al.*, 2005; Conley *et al.*, 2009*b*). Images were analyzed using Leica Confocal Software (Leica, V2.61) or ImageJ 1.45s (Schneider *et al.*, 2012). Chlorophyll, CFP, and YFP fluorescence was false colored red, cyan, and yellow, respectively.

### Western blots

For one protein extract, three leaf disc samples (9 mm) were collected from transiently transformed *N. benthamiana* plants at 4 dpi. Total soluble protein (TSP) was extracted and quantified as described by Conley *et al.* (2009*a*). For each sample, 10 μg of TSP were size separated by 10% SDS–PAGE. The proteins were probed with a primary anti-GFP antibody (Clontech catalog no. 632380; designed to recognize GFP and fluorescent variants including CFP) at a 1:5000 dilution. Subsequently, a secondary goat anti-mouse IgG (H+L) horseradish peroxidase-conjugated antibody (Bio-Rad catalog no. 170-6516) was used at a 1:3000 dilution. CFP fusion proteins were visualized with the enhanced chemiluminescence (ECL) detection kit (GE Healthcare, Mississauga, ON, Canada).

### Measurements and statistics

Stromule and chloroplast lengths were determined using the measuring tool from ImageJ 1.45s (Schneider *et al.*, 2012). Chloroplasts were measured in a straight line across their longest axis. Stromules were considered to be any extensions from chloroplasts that were >1 μm in length. Chloroplasts were analyzed for stromules only if they contained detectable TP-ADT2–CFP fluorescence. For non-linear stromules, several linear measurements were taken to account for bends and curves, and subsequently added together to provide a more accurate measurement of stromule length. Nuclear-localized ADT5–CFP was measured as a proportion of total cells exhibiting ADT5–CFP fluorescence. To determine the proportion of cells having ADT5–CFP within the nucleus, cells were analyzed only if ADT5–CFP was present in the cell/chloroplast. The proportion of chloroplasts with stromules, average stromule and chloroplast lengths, and proportion of cells with ADT5 in the nucleus were analyzed using one-way ANOVA (multiple comparisons) on GraphPad Prism 7.0.

## Results

### ADTs localize to stroma and stromules of chloroplasts

To determine the subcellular localization of ADTs, full-length *A. thaliana* ADTs were transiently expressed as CFP fusions in *N. benthamiana* leaves. *ADT* genes were cloned (Fig. 1B) upstream of the CFP-coding sequence to maintain the fluorescent tag even if the putative TP is cleaved *in planta* (Li and Chiu, 2010). ADTs were tagged with CFP, instead of GFP, to avoid emission spectra overlap with organelle markers tagged with YFP (Shaner *et al.*, 2005; Nelson *et al.*, 2007; Lu *et al.*, 2010). Leaves of 3- to 5-week-old *N. benthamiana* leaves were co-transformed with an *A. tumefaciens* strain harboring an *ADT–CFP* construct, and a strain encoding p19 to enhance recombinant protein expression (Voinnet *et al.*, 2003). For co-localization experiments, leaves were also co-transformed with *A. tumefaciens* carrying a plasmid-encoded organelle marker. ADT–CFP expression was confirmed by isolating total protein and performing western blots (Supplementary Fig. S2 at *JXB* online).

**Fig. 2.**
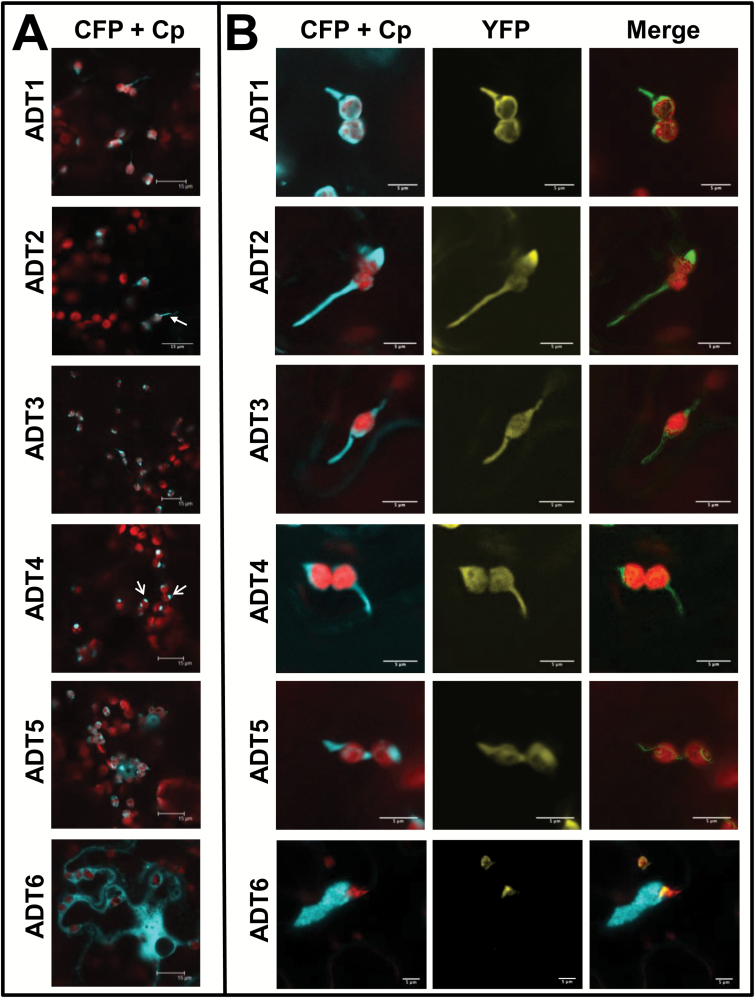
Subcellular localization of ADT–FP fusion proteins and co-localization with TP-ssRuBisCO–YFP. (A) ADT–CFP subcellular localization patterns. ADT1–ADT5 localized to stroma and to areas seemingly close to the chloroplast just outside of the autofluorescence signal generated by chlorophyll. They often appear either in thread-like structures (e.g. the arrow in ADT2) or globular structures (e.g. the arrows in ADT4). The ADT6–CFP pattern is distinctly different, showing a cytosolic distribution. Images were taken at a lower magnification to allow observation of the CFP signal relative to several chloroplasts. (B) Close-ups of ADT–CFP subcellular localization patterns in relation to TP-ssRuBisCO–YFP. In contrast to the chlorophyll autofluorescence, the TP-ssRuBisCO–YFP is a stroma-specific marker that visualizes all stroma-filled areas within the chloroplast including stromules. ADT1–ADT5 are found within the main body of chloroplast and in stromules, while ADT6 is found within the cytosol and does not co-localize with TP-ssRuBisCO–YFP.

As transformation controls, non-infiltrated tissue, pCB without an insert (empty vector; EV), and pCB infiltrated only with p19 were observed by confocal laser scanning microscopy. For all controls, only chlorophyll autofluorescence was detected and no fluorescence was observed in the CFP and YFP channels in the absence of fusion proteins (Supplementary Fig. S1). ADTs localized to the chloroplast stroma, but were also seen within thread-like structures (e.g. the arrow in ADT2) or as globular structures (e.g. arrows in ADT4) near the chloroplast, but the CFP signal did not directly overlap with chlorophyll autofluorescence (Fig. 2A). The shape and length of these structures were variable, ranging from short and globular to long and narrow protrusions from the chloroplast body. Unlike ADT1–ADT5, ADT6 did not localize to chloroplasts, but was mostly present in the cytosol (Fig. 2A, bottom panel).

We hypothesized that the thread-like and globular structures were stromules (Köhler and Hanson, 2000). To confirm that ADTs do localize to stromules, the *ADT–CFP* fusion constructs were co-expressed with the TP of the small subunit of RuBisCO fused to YFP (TP-ssRuBisCO–YFP). This construct is known to localize to the stroma, and therefore can be used to identify stromules (Nelson *et al.*, 2007). The fluorescence of CFP fusion proteins with ADT1–ADT5 overlapped with the fluorescence of TP-ssRuBisCO–YFP, which confirms that these ADTs are targeted to stromules within the chloroplasts (Fig. 2B). The fluorescence of ADT6–CFP did not overlap with the fluorescence of TP-ssRuBisCO–YFP as ADT6 is mostly found within the cytosol (Fig. 2B, bottom panel).

**Fig. 3.**
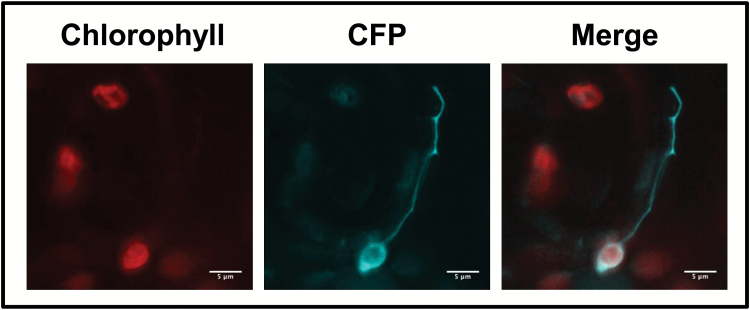
Localization of ADTs to the chloroplast is dependent on the transit peptide sequences. To test if the transit peptide sequences are sufficient for the transport of ADTs to the chloroplast, the first 99 amino acids of ADT2 (TP-ADT2–CFP) were expressed transiently in *N. benthamiana* leaves.

To determine if the TP domain of ADTs is responsible for chloroplast and stromule localization, the TP sequence was expressed as a TP-ADT2–CFP fusion protein and was detected in chloroplasts and stromules (Fig. 3). These data are consistent with the TP being sufficient to target ADT sequences to chloroplasts and stromules.

### ADT2 localizes to chloroplasts in a ring structure

**Fig. 4.**
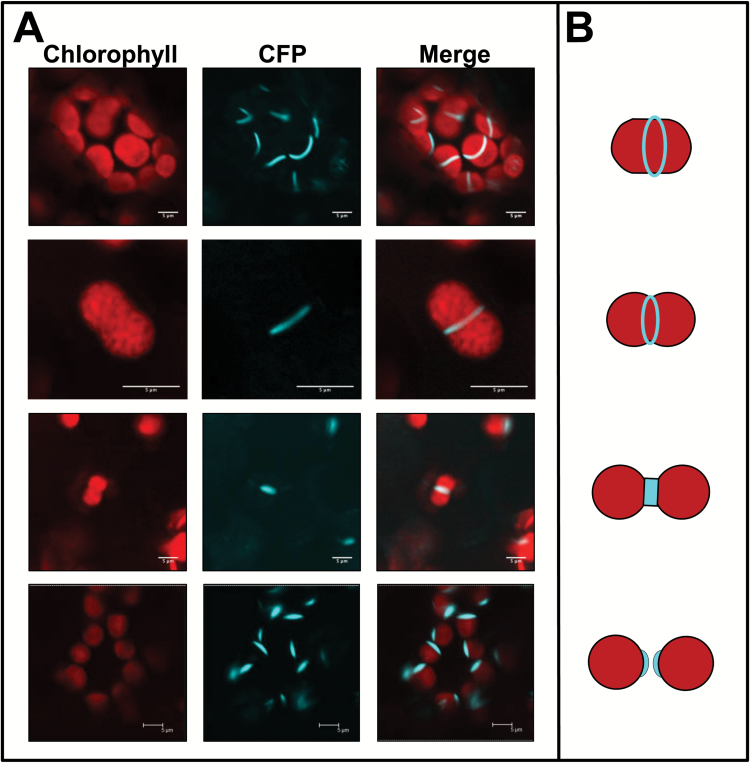
ADT2 forms structures consistent with chloroplast division rings. (A) In addition to being expressed within chloroplasts and stromules, ADT2 was also found to accumulate in places consistent with chloroplast division rings. The top panel shows ADT2 forming rings at the equatorial plate of the chloroplast. On occasion, ADT2 was found in the constriction zone of chloroplasts (two middle panels). In these cases, the chloroplasts have a distinct dumb-bell shape and the degree of indentation depends on how far the division process has proceeded. In addition, ADT2 accumulated in a spindle-like shape that tapers at chloroplast poles (bottom panel). This fusiform ADT2 accumulation was only found at one pole of the chloroplast and is distinct from a stromule pattern shown in Fig. 2. (B) Schematic of chloroplast division stages: from top to bottom, positioning of chloroplast division rings; slightly constricted chloroplast just prior to division; two daughter chloroplast following division. Analogous to the fluorescent images, chloroplasts are shown in red and the position of ring proteins in blue (adapted from Miyagishima, 2011).

ADT2–CFP displayed a unique localization pattern compared with the other ADTs (Fig. 4A). In chloroplasts with no apparent central constriction, ADT2–CFP localized to a band at the equatorial plane. Stacked confocal images showed that ADT2 formed a ring around the center of the chloroplast (data not shown). In elongated chloroplasts with a slight indentation, suggestive of an early chloroplast division stage, ADT2–CFP localized as a band around the middle of the elongation exactly at the point of indentation. In chloroplasts with a clear indentation, indicative of a later stage of division, ADT2–CFP was found at the site of constriction. ADT2 localization to the poles of chloroplasts was consistent with remnants of the division ring on daughter chloroplasts (Miyagishima, 2011). These ADT2 localization patterns are strikingly similar to those of proteins that are involved in chloroplast division, a process requiring placement of multiple proteinaceous rings followed by constriction that partitions the chloroplast into two equal-sized daughter chloroplasts (Fig. 4B; Miyagishima, 2011).

To initiate division, chloroplasts have to reach a certain size (Pyke, 1999). Therefore, we argue that chloroplasts with ADT2–CFP at the equatorial plane should be the largest as they are in the process of dividing. Conversely, chloroplasts with ADT2–CFP at their pole should be the smallest as they have just recently divided. In addition, these two classes should have little variation in size as they represent distinct stages in chloroplast development. In contrast, growing chloroplasts should vary in size as they encompass all division stages. While chloroplast volume would be the most accurate way to measure chloroplast size, it is difficult to determine. Therefore, chloroplast size was measured as the length of a chloroplast across its longest axis. Chloroplasts from uninfiltrated *N. benthamiana* plants were used to determine the average size of a chloroplast because they should contain chloroplasts at many different developmental stages, and thus different sizes. Chloroplast sizes measured in three uninfiltrated plants had an average length of 5.1 μm (Table 2). In chloroplasts with ADT2–CFP present on a pole, the average length of these chloroplasts was significantly shorter (*P*<0.05), at 4.2 μm (Table 2). Lastly, chloroplasts with ADT2–CFP localized at the equatorial plane were significantly longer, at 6.7 μm (*P*<0.05; Table 2). As predicted, the SD from the mean was larger for chloroplasts from uninfiltrated plants, consistent with a mixed population of chloroplasts, compared with chloroplasts with ADT2–CFP at a pole or at the equatorial plane (Table 2). These data support the hypothesis that the ADT2 localization patterns we observed are consistent with different chloroplast division stages.

**Table 2.** Comparison of chloroplast lengths

**Type of chloroplast**	**No. of plants**	**No. of chloroplasts**	**Length (µm**)^*a*^	**SD**
Uninfiltrated	3	68	5.1	1.12
With polar ADT2	3	75	4.2*	0.60
With equatorial ADT2 ring	5	35	6.7*	0.85

^*a*^ The length of the chloroplast was measured at its longest axis.

* Significantly different from the chloroplasts in the uninfiltrated control (*P*<0.05) as determined by a *t*-test.

### *A single amino acid change in ADT2 affects chloroplast morphology and FtsZ2 localization in* A. thaliana

**Fig. 5.**
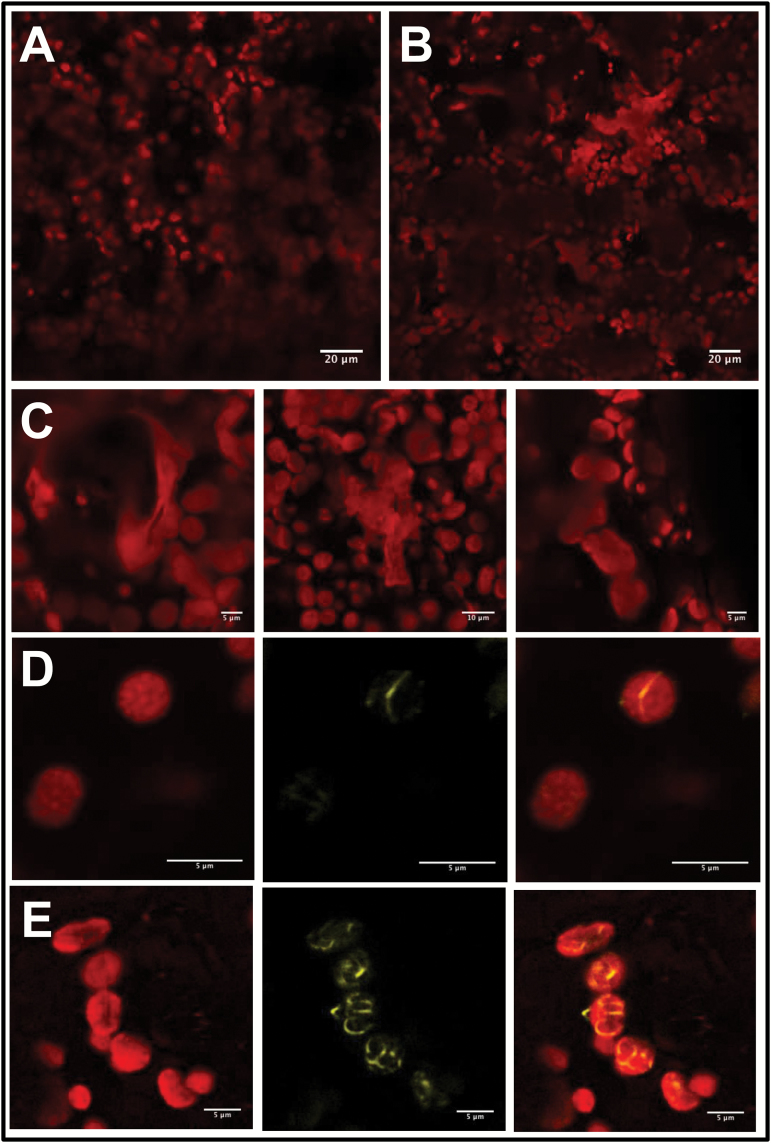
Chloroplast morphology and FtsZ2–YFP localization is affected by a point mutation in *ADT2*. (A) Chloroplasts in wild-type *A. thaliana* Col-0. (B) Chloroplasts in *adt2-1D A. thaliana* mutants. (C) Close-ups of chloroplasts observed in *adt2-1D* to show the heterogeneity in shape and size. (D) Transiently expressed FtsZ–YFP in wild-type Col-0 localizes as expected to a single ring at the equatorial plane. (E) In contrast, FtsZ2–YFP localizes as long spiralling filaments within *adt2-1D* chloroplasts. (D, E) Images of chlorophyll fluorescence (left) and FtsZ2–YFP (middle) are shown separately and merged (right).

Mutations in genes encoding components of the chloroplast division machinery result in changes to chloroplast morphology (Pyke, 1999). If ADT2 has a role in chloroplast division, it is reasonable to expect that an *adt2* mutant will have distorted chloroplasts. Unlike for other *ADT* genes, no *A. thaliana* T-DNA insertion line that abolishes *ADT2* mRNA production exists (Corea *et al.*, 2012*b*). However, a point mutation within the coding sequence of the ACT regulatory domain of *ADT2* (*adt2-1D*) has been documented in which conversion of a serine to an alanine prevents allosteric inhibition of the enzyme (Huang *et al.*, 2010). To determine if *adt2-1D* affects chloroplast morphology, homozygous *adt2-1D* plants were examined by confocal microscopy and compared with wild-type Col-0 plants of identical age (Fig. 5A, B). Chloroplasts in *adt2-1D* plants differed greatly in appearance, and were highly heterogeneous in size and shape (Fig. 5C). This contrasted with chloroplasts in wild-type Col-0, which were ovoid in shape and relatively uniform in size. Although many *adt2-1D* chloroplasts were clearly affected by the mutation, wild-type appearing chloroplasts can still be observed, suggesting a partial loss of ADT2 function.

FtsZ is a tubulin-like protein that is a central component of the chloroplast division apparatus (Vitha *et al.*, 2001; TerBush *et al.*, 2013). There are two FtsZ proteins (FtsZ1 and FtsZ2), which assemble to form the earliest known division ring, the Z-ring within the stroma. As the localization of FtsZ in chloroplast division mutants has been used to provide insight into the function of putative division proteins (Vitha *et al.*, 2003; Glynn *et al.*, 2007; Fujiwara *et al.*, 2008; Glynn *et al.*, 2009; Nakanishi *et al.*, 2009*a*), we were interested to determine if FtsZ localization was affected in *adt2-1D* plants. Thus we generated an *FtsZ2–YFP* fusion construct and expressed it in *adt2-1D* plants. Expression of FtsZ2–YFP in wild-type Col-0 leaves led to the formation of the expected single ring (Fig. 5D). In contrast, FtsZ2–YFP in *adt2-1D* plants was less organized and formed what appeared to be spirals or multiple rings (Fig. 5E). These results demonstrate that deregulation of ADT2 abolishes proper placement of FtsZ2, further supporting an involvement of ADT2 in chloroplast division.

### ADT5 is found in the nucleus

**Fig. 6.**
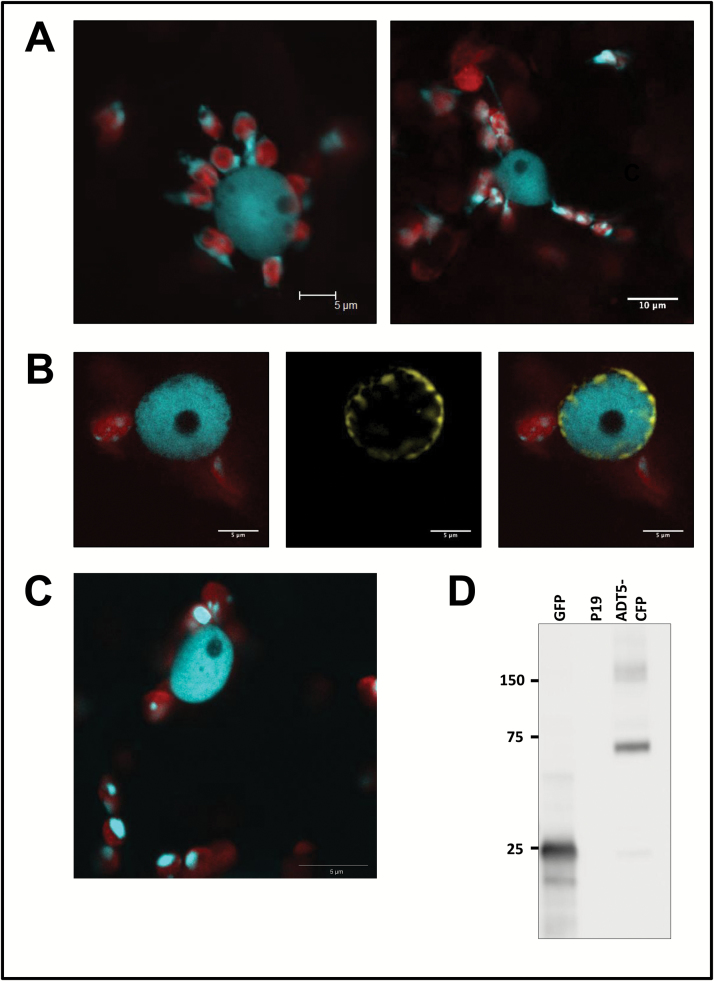
ADT5 is found in the nucleus. ADT5–CFP proteins are unique as they are the only full-length ADT proteins that were found in the nucleus. (A) Nuclei show a close association with chloroplasts (left) or with stromules of chloroplasts (right). Both images show ADT5–CFP within nuclei. (B) Co-localization of ADT5–CFP with NUP1–YFP. To determine if ADT5–CFP localizes to the nucleus, it was co-expressed with NUP1–YFP in *N. benthamiana*. Images of chlorophyll fluorescence and ADT5–CFP are shown merged (left). NUP1–YFP is shown alone (middle) and merged with ADT5–CFP and chlorophyll fluorescence (right). NUP1–YFP localizes to the nuclear membrane and surrounds ADT5–CFP, confirming that it localizes to the nucleus. (C) ADT5–CFP transiently expressed with its native *ADT5* promoter also localizes to the nucleus. (D) Western blot of ADT5–CFP (calculated size 73.9 kDa) expressed with its native promoter and visualized with a GFP antibody is detected at its expected size. As negative controls, proteins isolated from leaves transformed with GFP (25 kDa) and p19 are shown. Total soluble protein was isolated from transiently transformed leaves, and 10 μg of total soluble protein was size separated by 10% SDS–PAGE. Sizes of the protein ladder are given in kDa.

In addition to its chloroplast localization, only ADT5–CFP was also detected in nuclei at ~4–5 dpi (Figs 2A, 6A). To confirm this finding, ADT5–CFP was co-infiltrated with a YFP fusion to the nuclear marker NUP1, a component of the nuclear pore complex in *A. thaliana* that was previously shown to localize to the nuclear membrane (Lu *et al.*, 2010). Confocal imaging determined that NUP1–YFP localized around ADT5–CFP (Fig. 6B). As NUP1–YFP localizes to the nuclear membrane, this result confirms that ADT5–CFP is contained within the nucleus and localizes uniformly throughout the nucleoplasm. As these results were obtained with constructs using a CaMV *35S* promoter, we repeated the experiment and expressed ADT5–CFP under control of its native promoter (Fig. 6C) and confirmed the nuclear localization pattern. To ensure that the observed nuclear localization is not due to a smaller diffusible cleavage product or to a particularly high level of ADT5 compared with other ADTs, we performed western blots (Fig. 6D; Supplementary Fig. S2). The blot containing all ADTs expressed under the control of the CaMV *35S* promoter shows a low level of cleavage in all lanes. However, more cleavage product is seen for ADT1 and ADT3 compared with ADT5, while no nuclear localization is observed with either ADT1 or ADT3. The blot showing ADT5–CFP under the control of its native promoter (Fig. 6D) shows a shadow band of the size of CFP, and yet a very clear nuclear localization is evident (Fig. 6C). These results indicate that the nuclear localization pattern is a *bona fide* ADT5 localization. There are additional bands of higher molecular weight (Fig. 5D; Supplementary Fig. S2) that might correspond to ADT dimers or even higher multimers.

**Fig. 7.**
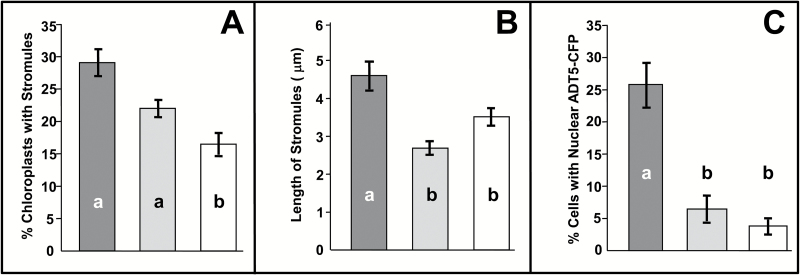
The presence of ADT5 in the nucleus is affected by the ability to form stromules. To determine if nuclear localization of ADT5 is dependent on stromules, plants were co-infiltrated with TP-ADT2–CFP (A and B) as a control or ADT5–CFP (C) and an empty vector (dark gray), dominant negative myosin XI-2 (dnMyoXI-2; light gray) and myosin XI-K (dnMyoXI-K/GTD; white), respectively. (A) Percentage of chloroplasts having stromules. Chloroplasts were analyzed if they contained any visible TP-ADT2–CFP fluorescence and were determined to have a stromule if the projection was longer than 1 μm. In total 554, 395, and 579 chloroplasts were analyzed from plants transformed with an empty vector, dnMyoXI-2, and dnMyoXI-K/GTD, respectively. (B) Average length of stromules. A total of 166, 93, and 91 stromules were measured from plants transformed with an empty vector, dnMyoXI-2, and dnMyoXI-K/GTD, respectively. (C) Nuclear localization of ADT5–CFP. Cells were analyzed for CFP fluorescence in the nucleus only if any ADT5–CFP fluorescence was detectable. A total of 131, 190, and 358 cells were analyzed from plants transformed with an empty vector, dnMyoXI-2, and dnMyoXI-K/GTD, respectively. Each experiment was performed on three independent occasions. Significant differences (*P*<0.001) as determined by a one-way ANOVA (multiple comparisons) are indicated by different letters. Averages ± SE of the mean are plotted.

Furthermore, we often observed ADT5–CFP-containing nuclei surrounded by chloroplasts that appeared to be connected to the nucleus through stromules (Fig. 6A), suggesting that ADT5 nuclear localization depends on stromule-mediated transport. To test if stromule formation affects the nuclear localization of ADT5, we transiently expressed myosin XI tail domains (*dnMyoXI-2* and *dnMyoXI-K/GTD*) as they were previously shown to inhibit stromule formation through a dominant negative effect on wild-type myosin XI (Avisar *et al.*, 2008; Natesan *et al.*, 2009). To ensure that these treatments inhibit stromules, several control transformations were performed. To visualize stromules in control transformations, TP-ADT2–CFP was used, as it easily visualizes stromules and therefore provides a very sensitive marker to detect stromule inhibition. TP-ADT2–CFP was co-infiltrated into *N. benthamiana* leaves with an empty pCB vector, a *dnMyoXI-2* construct, or a *dnMyoXI-K/GTD* construct (Avisar *et al.*, 2008), and the number of chloroplasts having stromules (Fig. 7A) and the length of stromules formed (Fig. 7B) were determined. In plants transformed with an empty vector, 28.7% of chloroplasts had stromules (Fig. 7A) and the average length of these was 4.6 μm (Fig. 7B). Infiltration with dnMyoXI-2 decreased the percentage of chloroplasts with stromules (22.2%; Fig. 7A) compared with that of the control, and significantly reduced (*P*<0.001) the average length of stromules to 2.7 μm (Fig. 7B). Treatment with dnMyoXI-K/GTD also caused a significant decrease (*P*<0.001) in the percentage of chloroplasts with stromules (17.3%; Fig. 7A) and significantly decreased (*P*<0.001) the average length of stromules to 3.5 μm (Fig. 7B). These results confirm that both myosin domains affect stromule formation.

To determine if the ability to form stromules affects nuclear localization of ADT5, *ADT5–CFP* was co-expressed with the empty pCB vector, *dnMyoXI-2*, or *dnMyoXI-K/GTD* (Fig. 7C). The extent of ADT5–CFP nuclear localization was expressed as a percentage of cells containing CFP fluorescence. Co-infiltration with the empty vector control showed that 25.9% of cells had ADT5–CFP fluorescence visible in the nucleus (Fig. 7C). In contrast, co-infiltration with *dnMyoXI-2* or *dnMyoXI-K/GTD* showed that ADT5–CFP was detected in the nucleus in only 7.1% and 4.3% of cells, respectively (Fig. 7C), both significant reductions (*P*<0.001) from the control. These data demonstrate that ADT5–CFP nuclear localization is decreased by the same conditions shown to decrease stromule formation.

### *ADTs in* A. thaliana

Transient transformations using agroinfiltration are widely used in *N. benthamiana* but have traditionally been difficult in *A. thaliana* (Wroblewski *et al.*, 2005). We were able to transform *A. thaliana* reliably by growing plants with 20 mM l-ascorbic acid, which seemed to decrease necrosis of leaves associated with agroinfiltration. As it was not the focus of this study, the reason for this was not addressed. However, as l-ascorbic acid can scavenge damaging reactive oxygen species (Gallie, 2013), the increased tolerance of *A. thaliana* to agroinfiltration may be due to a decrease in oxidative stress.

**Fig. 8.**
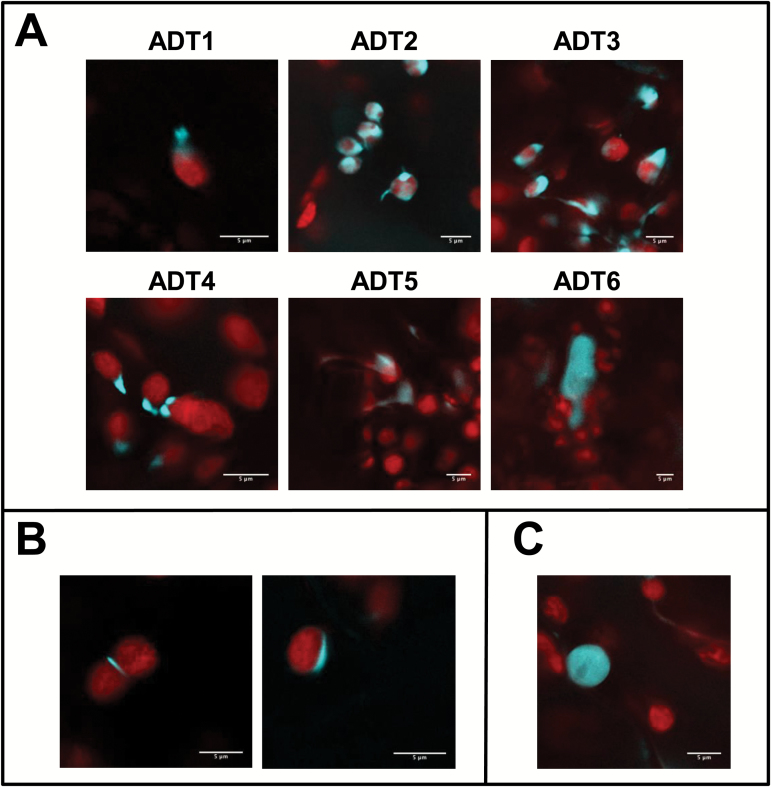
ADT localization to the stroma and stromules, the chloroplast equatorial plane, and the nucleus can also be detected in *A. thaliana*. To test if the ADT patterns determined in *N. benthamiana* reflect expression in *A. thaliana*, all six ADT–CFP fusion proteins were transiently expressed in *A. thaliana* Col-0. All images show a merge of chlorophyll and CFP fluorescence. (A) ADT1–CFP through ADT5–CFP localize to stroma and structures resembling stromules of varying shapes and lengths, with varying levels of fluorescence in the stroma. ADT6–CFP localizes outside of chloroplasts in the cytosol. (B) Chloroplast division patterns for ADT2–CFP. (C) Nuclear localization of ADT5–CFP.

*ADT–CFP* fusion genes were transiently expressed in *A. thaliana* to confirm that localization in a heterologous host reflected the situation in the native environment (Fig. 8). As in *N. benthamiana*, all ADT–CFPs in *A. thaliana*, with the exception of ADT6–CFP, which appeared in the cytosol, exhibited stroma and stromule-like patterns (Fig. 8A). Similarly, the unique localization patterns of ADT2–CFP and ADT5–CFP were observed in *A. thaliana*, localizing to the equatorial plane and poles of chloroplasts (Fig. 8B), and to nuclei (Fig. 8C), respectively. This finding was important as it verifies that the findings in *N. benthamiana* are not artifacts.

## Discussion

### Phenylalanine biosynthesis and stromules

ADTs were seen to localize to thread-like structures seemingly outside of the region of chlorophyll autofluorescence (Fig. 2A). Co-localization of ADT–CFP fluorescence with the stromule marker TP-ssRuBisCO–YFP (Nelson *et al.*, 2007) confirmed that all ADTs except ADT6 localized to stromules (Fig. 2B). Prior to our study, *A. thaliana* ADTs were found uniformly throughout the stroma of chloroplasts, with no indication of stromule localization (Rippert *et al.*, 2009). However, the said study used protoplasts, which represent dedifferentiated cells that are also in a state of stress (Genschik *et al.*, 1992; Reyes *et al.*, 2010).

Stromules are dynamic structures that range in size and shape from short beak-like projections to long and elaborate tubules (Köhler and Hanson, 2000; Gunning, 2005; Gray, 2013). The function of stromules has been the subject of debate, but the idea that they increase transport of compounds synthesized within plastids to other areas of the cell is now generally accepted (Hanson and Sattarzadeh, 2013). The presence of biosynthetic enzymes, such as ADTs, in stromules is consistent with this hypothesis. Phenylalanine, the product of ADT enzymatic activity, is required within the cytosol for the synthesis of proteins and as a precursor for phenylpropanoids such as lignins and flavonoids (Fraser and Chapple, 2011). It stands to reason that stromules could have a high concentration of phenylalanine as a result of ADT activity, providing an effective means of increasing phenylalanine export into the cytosol. Interestingly, abiotic stressors, such as drought and salt stress, known to induce stromules (Gray *et al.*, 2012), are also associated with increased flavonoid levels in leaves (Agati *et al.*, 2011; Mewis *et al.*, 2012), and genes encoding flavonoid biosynthetic enzymes are up-regulated in response to salinity-induced stress (Walia *et al.*, 2005). For example, PHENYLALANINE AMMONIA LYASE1 (PAL1) uses phenylalanine as a substrate to catalyze the first step of phenylpropanoid biosynthesis in the cytosol (Walia *et al.*, 2005; Fraser and Chapple, 2011). Under conditions of salt stress, *PAL1* up-regulation coincides with the formation of stromules, suggesting that these two events might be linked and that both events contribute to an increased transport of phenylalanine to the cytosol.

ADTs are not the only enzymes that have been associated with stromules. Other examples include geranylgeranyl diphosphate synthase (GGPS), which synthesizes geranylgeranyl diphosphate in the stromules of chloroplasts, and the compound is required in the cytosol as part of isoprenoid metabolism (Thabet *et al.*, 2012), or RuBisCO and an aspartate aminotransferase (ASP5), which are present in stromules and capable of moving between plastids via stromules when expressed as GFP fusion proteins (Kwok and Hanson, 2004). This allows for the speculation that chloroplastic enzymes that synthesize molecules required in the cytosol preferentially localize to stromules as these might facilitate metabolite export.

*In silico* analysis of *A. thaliana* ADT sequences using ChloroP (Emanuelsson *et al.*, 1999) predicted that their N-terminal sequences were likely to encode TPs directing the enzymes to the chloroplast. We present data that confirm the *in silico* prediction for ADT2, as the N-terminal portion was necessary and sufficient to allow direct import into chloroplasts and specifically stromules (Fig. 3A, B). These data also corroborate observations made for all three petunia ADTs, where the N-terminal portion of ADTs directed GFP fusion proteins to chloroplasts (Maeda *et al.*, 2010).

### ADT2 and a role in chloroplast division

Aside from its inclusion in stroma and stromules, ADT2 localized as a ring around the equatorial plane or at the poles of chloroplasts (Figs 4A, 8B). The similarity of these patterns to those of chloroplast division proteins (Miyagishima, 2011) during and after division led to an investigation of a possible second, non-enzymatic role for ADT2 in chloroplast division. Since chloroplast division is regulated by size (Pyke, 1999), we reasoned that chloroplasts with either the equatorial or polar localization patterns would be larger and smaller, respectively, than average sized chloroplasts. This was confirmed upon comparing chloroplast lengths across their longest axis (Table 2). Additionally, the SD of chloroplast lengths varied. It was lowest in chloroplasts with ADT2 at the equator or at a pole, in agreement with these chloroplasts being in very distinct phases, either just prior to or post-division, and therefore very similar in size. This was in contrast to chloroplasts from uninfiltrated plants, which are comprised of chloroplasts in all division states. Therefore, our results suggest that ADT2 localizes to the division plane early in the division process and remains there throughout the duration of constriction and separation into daughter organelles. Similarly, known division proteins such as FtsZ and ACCUMULATION AND REPLICATION OF CHLOROPLASTS 6 (ARC6) assemble at the equatorial plane in the process leading to constriction and division (Vitha *et al.*, 2001; 2003).

The striking similarities between ADT2 and other chloroplast division proteins prompted observation of chloroplasts in *adt2* mutant plants. Interestingly, no T-DNA insertion knockout lines that abolish *ADT2* mRNA are available (Corea *et al.*, 2012*b*). This makes ADT2 unique and raises the possibility that an *adt2* knockout is lethal. However, plants homozygous for the *adt2-1D* point mutation have been documented (Huang *et al.*, 2010). The appearance of *adt2-1D* chloroplasts was variable and the presence of misshapen and heterogeneous chloroplasts is consistent with previous descriptions of chloroplast morphology in division mutants (Pyke and Leech, 1992; Colletti *et al.*, 2000; Glynn *et al.*, 2009; Nakanishi *et al.*, 2009*b*). In the *adt2-1D* plants, chloroplasts that appear wild type can still be observed. This infers that the single amino acid substitution probably does not abolish ADT2’s function, but impairs it. During chloroplast division, FtsZ forms the first known division ring within the stroma (TerBush *et al.*, 2013). Given the central role that FtsZ proteins play in division, we reasoned that FtsZ localization should be affected in *adt2-1D* chloroplasts if ADT2 is a chloroplast division protein. Expression of an *FtsZ2–YFP* fusion construct in *adt2-1D* plants revealed long and spiraling FtsZ2–YFP filaments throughout the chloroplast stroma. Although FtsZ2–YFP was overexpressed in our study, in wild-type Col-0 chloroplasts the fusion protein localized as expected as a single equatorial ring. We propose that the abnormal appearance of FtsZ2–YFP within *adt2-1D* chloroplasts suggests that ADT2 regulates FtsZ positioning. However, we cannot ignore the possibility that elevated phenylalanine levels in *adt2-1D* (up to 160-fold compared with the wild type; Huang *et al.*, 2010) are at least in part responsible for the observed changes.

It is intriguing to note that not all ring proteins have been identified. Although FtsZ, one of the inner rings, and ARC5 (dynamin), one of the outer rings, have been known for some time (Vitha *et al.*, 2001; Gao *et al.*, 2003), the identity of the outer plastid-dividing (PD) ring (Yoshida *et al.*, 2010), polyglucan filaments, has just recently been suggested, and the composition of the inner PD ring is still unknown. The realization that enzymes can have a second unrelated, non-enzymatic function, even as part of cellular structural components, is discovered more and more frequently (Huberts and van der Klei, 2010; MoonProt Database: www.moonlightingproteins.org, last accessed 13 February 2017). A good example of this is the *Physcomitrella patens* enzyme presenilin, the catalytic unit for γ-secretase, which has an independent function in the cytoskeletal network (Khandelwal *et al.*, 2007). However, additional studies will be required to determine the precise relationship between ADT2, phenylalanine levels, chloroplast division, and other components of the chloroplast division machinery.

### ADT5 and the nucleus

Similar to ADT2, ADT5 has an additional unique localization pattern and was clearly observed in the nuclei of both *N. benthamiana* and *A. thaliana* (Fig. 6A, C). Proteins with dual plastid and nuclear localization may be significant in the context of retrograde signaling. While retrograde signaling traditionally refers to chemical messengers that are released from plastids and affect nuclear gene expression (Inaba *et al.*, 2011), it is becoming apparent that proteins within the chloroplast can also act as retrograde signals (Isemer *et al.*, 2012; Krause *et al.*, 2012). One such protein is WHIRLY1 from *A. thaliana*, which can move directly from plastids to the nucleus (Isemer *et al.*, 2012). In plastids, WHIRLY1 contributes to plastid genome stability by preventing illegitimate recombination (Maréchal *et al.*, 2009). In the nucleus, it acts as a transcriptional activator of pathogen response genes (Isemer *et al.*, 2012), consistent with the increased pathogen susceptibility associated with decreased WHIRLY1 DNA binding ability (Desveaux *et al.*, 2005). Whether ADT5 has a role in retrograde signaling is currently unknown. In addition, many other enzymes with diverse functions have been reported in both plastids and nuclei, such as phosphate-isopentyltransferase 3, an enzyme involved in cytokinin biosynthesis (Galichet *et al.*, 2008; Krause *et al.*, 2012), CDT1, a kinase required in cell cycle regulation (Raynaud *et al.*, 2005), and a dihydrofolate reductase required for nucleotide metabolism (Luo *et al.*, 1997), but often the nuclear role of these enzymes is ill defined.

While a direct mechanism of protein transport between plastids and the nucleus through stromules is a hypothetical mode (Krause *et al.*, 2012), they have been shown to interconnect plastids (Köhler *et al.*, 1997; Kwok and Hanson, 2004; Hanson and Sattarzadeh, 2013). Chloroplasts with stromules containing ADT5–CFP often appeared to connect directly with the nucleus (Fig. 6A). Expression of dominant negative forms of myosin XI were found to inhibit stromules (Fig. 7A, B) and also significantly reduce ADT5–CFP localization to the nucleus (Fig. 7C), providing indirect evidence of stromule-mediated nuclear transport. We are aware that there are alternative interpretations of these results, as myosin XI is involved in other processes including movement of organelles (Avisar *et al.*, 2008) and cytoplasmic streaming (Shimmen and Yokota, 2004). Regardless of this, the appearance of stromules directly connecting to nuclei (Fig. 6A) makes the possibility of a stromule-mediated nuclear transport system intriguing.

Currently, the role that ADT5 plays in the nucleus is unknown. It is conceivable that it acts as a transcriptional regulator of *ADT* genes or other genes within the same biosynthetic pathway. There is precedence for an enzyme to act as a transcriptional regulator of functionally related genes. For example, *A. thaliana* HEXOKINASE1 (HXK1) is involved in glucose metabolism in mitochondria, but also localizes to the nucleus where it forms part of a protein complex affecting transcription of genes involved in glucose signaling (Cho *et al.*, 2006). Furthermore, in the budding yeast *Saccharomyces cerevisiae*, ARG5,6, an enzyme involved in arginine biosynthesis, is able to bind DNA directly and regulate gene expression (Hall *et al.*, 2004). Interestingly, mutant analysis has shown that loss of ADT5 activity cannot be compensated for by other ADTs and is the only single *ADT* knockout with a visible phenotype (Corea *et al.*, 2012*b*), consistent with a unique nuclear role for ADT5.

### ADT2 and ADT5: moonlighting proteins

The term ‘moonlighting protein’ was coined to describe proteins that perform multiple autonomous and often unrelated functions without these functions being partitioned into different domains of the protein or resulting from alternative splicing or gene fusion (reviewed in Jeffery, 1999, 2013). Since the description of the first moonlighting proteins, the number of documented moonlighting proteins has increased to ~300 (Jeffery, 2013; MoonProt Database: www.moonlightingproteins.org). Enzymes are common among moonlighting proteins and many have additional non-enzymatic functions, including roles as structural components and as regulators of transcription or translation (Jeffery, 2013). For example, in *Tetrahymena*, a citrate synthase acts as an enzyme in mitochondria while in the cytosol it can polymerize to form 14 nm filaments and then act as a cytoskeletal protein (Kojima *et al.*, 1997). We propose that ADT2 and ADT5 are moonlighting proteins. In the current study we provide evidence that ADT2, with demonstrated arogenate dehydratase activity (Cho *et al.*, 2007), can form rings around chloroplasts similar to FtsZ or ARC5 as part of their role in chloroplast division (Vitha *et al.*, 2001; Gao *et al.*, 2003). The dual localization of ADT5 to chloroplasts and nuclei suggests that ADT5 has an additional role in the nucleus, possibly in transcriptional regulation. As the entire ADT enzyme family catalyzes the final step in phenylalanine biosynthesis, we expect this to be a key regulatory step.

Moonlighting proteins appear to be ubiquitous in nature, with documented examples in simple single-cell organisms, such as archaea and bacteria, and complex eukaryotes including plants and animals. It seems likely that moonlighting proteins are evolutionarily advantageous, ensuring that the number of genes in a genome does not limit the number of functions they are capable of performing. Many moonlighting proteins are ancient in terms of their evolutionary history, giving them ample time to adapt to a second role (Jeffery, 2013). Analyzing the subcellular localization of *A. thaliana* ADTs shows that the function of enzymes is far more complex than previously realized.

## Supplementary data

Supplementary data are available at *JXB* online.

Fig. S1. Negative controls.

Fig. S2. Western blots showing expression of transiently expressed ADTs.

## Supplementary Material

Supplementary_Figure1_2

## Abbreviations:

ACT: aspartokinase–chorismate mutase–TyrA
ADT: arogenate dehydratase
CFP: cyan fluorescent protein
dpi: days post-infiltration
EV: empty vector
GFP: green fluorescent protein
FtsZ: filamentous temperature sensitive Z
PAT: prephenate aminotransferase
PDT: prephenate dehydratase
PP: phenylpyruvate
PPAT: phenylpyruvate aminotransferase
PTGS: post-transcriptional gene silencing
TP: transit peptide
YFP: yellow fluorescent protein.
