# Supplementary material for: Subcellular localization of Arabidopsis arogenate dehydratases suggests novel and non-enzymatic roles
Source: J Exp Bot. 2017 Mar 10;68(7):1425–40. doi: 10.1093/jxb/erx024 (PMC5444438; doi:10.1093/jxb/erx024)
Supplement: Supplementary_Figure1_2 [file erx024_suppl_Supplementary_Figure1_2.pdf]

Subcellular Localizations of *Arabidopsis* Arogenate Dehydratases Suggest Novel and Non-Enzymatic Roles

Running Title: Subcellular Localizations of Arogenate Dehydratases

Crystal D. Bross, Travis R. Howes, Sara Abolhassani Rad, Ornela Kljakic, Susanne E. Kohalmi

Fig. S1. Negative controls.

To ensure that the CFP and YFP signals from fusion proteins are due to the presence of the desired fluorophore, several controls were performed. *N. benthamiana* mesophyll cells were excited with a 405 nm blue diode laser for CFP and chlorophyll, and subsequently a 514 nm argon laser for YFP. Fluorescence was collected from 440 to 485 nm for CFP, from 540 to 550 nm for YFP and from 630 to 690 nm for chlorophyll. Fluorophore channels were merged using ImageJ (Schneider *et al.*, 2012).

(A) Untransformed leaf tissue.

(B) Leaf tissue transformed with pCB (empty vector) only.

(C) Leaf tissue transformed with p19 and pCB with no insert.

Fig. S2. Western Blots showing expression of transiently expressed ADTs.

Western Blot showing the six ADT-CFP fusion proteins transiently expressed in *N. benthamiana* leaves and visualized with a GFP-antibody. The calculated sizes for the ADT fusion proteins are: ADT1 (71.5 kDa), ADT2 (70.0 kDa), ADT3 (74 kDa), ADT4 (73.8 kDa), ADT5 (73.8 kDa), and ADT6 (72.7). As negative controls, proteins isolated from leaves transformed with GFP (25 kDa) and P19 are shown. Total soluble protein was isolated from transiently transformed leaves and 10 µg of total soluble protein was size separated on a 10% SDS-PAGE gel. Sizes of protein ladder are given in KDa.

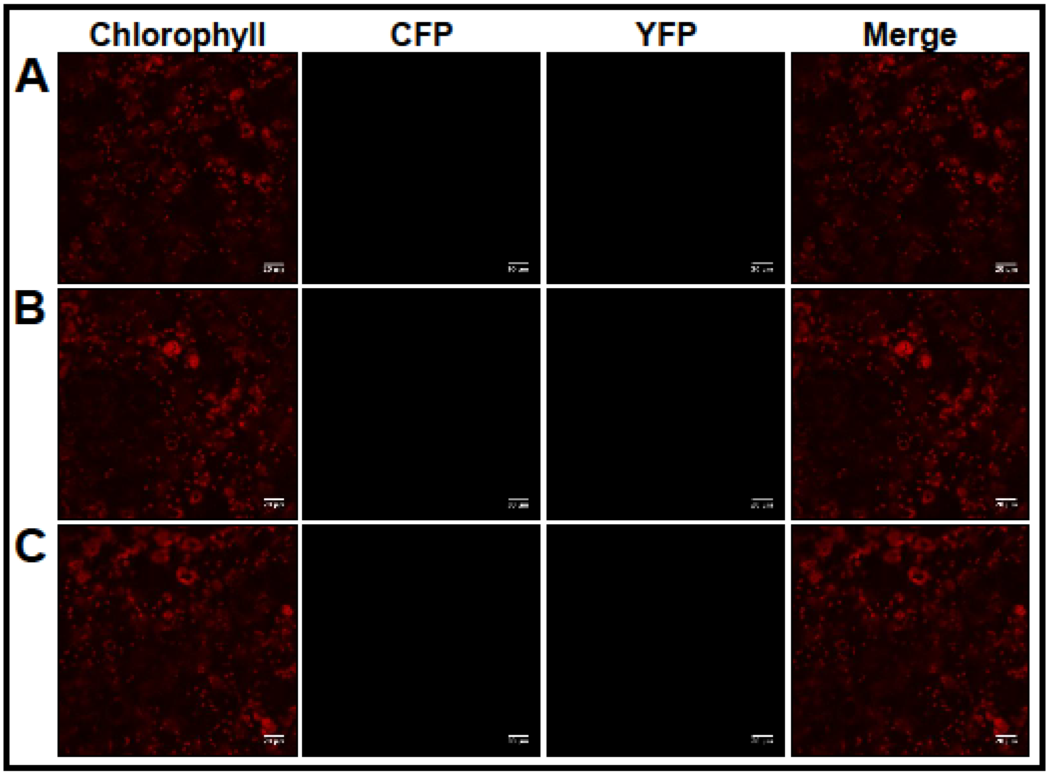

Figure S1

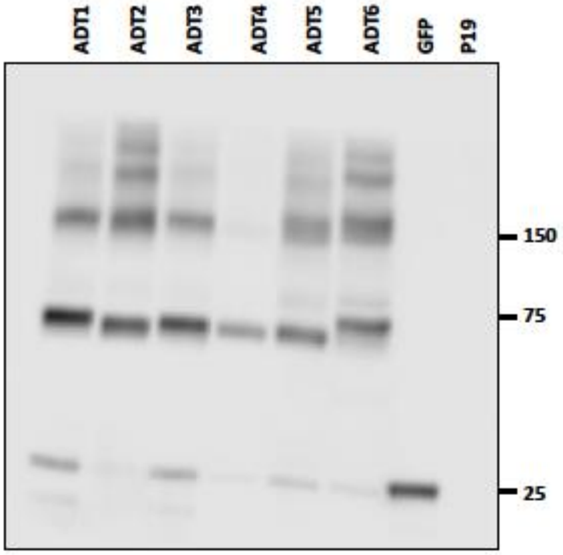

Figure S2
